# Supplementary material for: Ultrasound Measurement of Tumor-Free Distance from the Serosal Surface as the Alternative to Measuring the Depth of Myometrial Invasion in Predicting Lymph Node Metastases in Endometrial Cancer
Source: Diagnostics (Basel). 2021 Aug 14;11(8):1472. doi: 10.3390/diagnostics11081472 (PMC8392068; doi:10.3390/diagnostics11081472)
Supplement: Supplementary file 1 [file diagnostics-11-01472-s001.zip › Diagnostics_Figure S2.pdf]

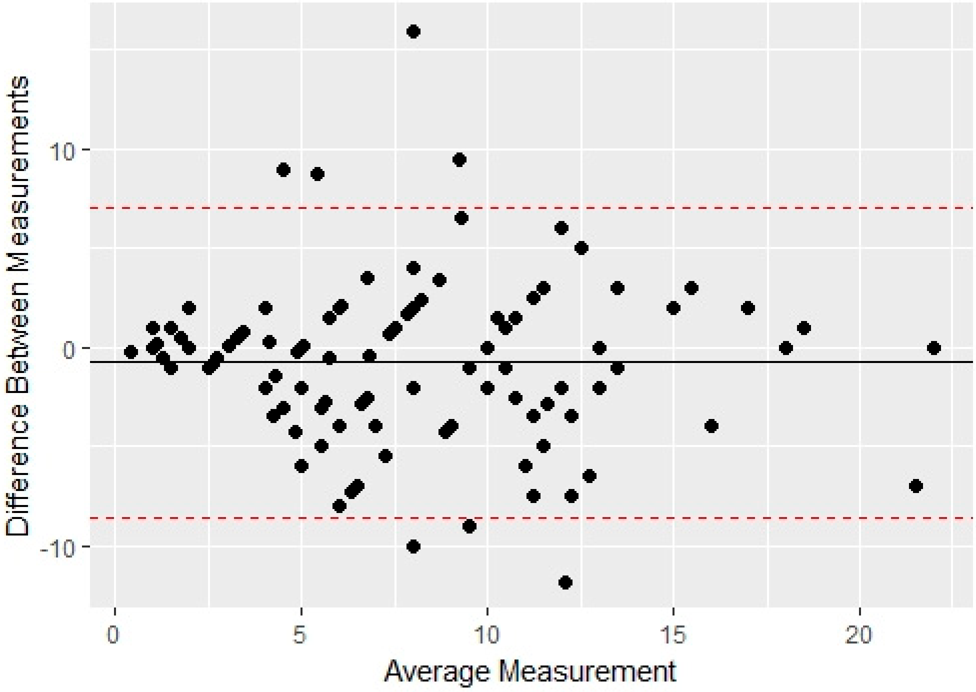

Interclass correlation coefficient (ICC) = 0,676, 95% CI (0,564 – 0,764)

Figure S2. Concordance between uTFD and pTFD (Bland-Altman plot).
