# Supplementary material for: Ultrasound Measurement of Tumor-Free Distance from the Serosal Surface as the Alternative to Measuring the Depth of Myometrial Invasion in Predicting Lymph Node Metastases in Endometrial Cancer
Source: Diagnostics (Basel). 2021 Aug 14;11(8):1472. doi: 10.3390/diagnostics11081472 (PMC8392068; doi:10.3390/diagnostics11081472)
Supplement: Supplementary file 1 [file diagnostics-11-01472-s001.zip › Diagnostics_Figures S3-S4.pdf]

Figures S3-S4

Regarding uMI (less or equal- or over 1/2 or 50%) differences between survival curves are not statistically significant (Log-rank test, p=0.5949) (Figure S3a). Differences between survival curves according to uTFD (<5.2 mm, >= 5.2 mm) are not statistically significant (log-rank test, p=0.9225) (Figure S3b). Differences between recurrence-free survival curves regarding uMI were not statistically significant (log-rank test, p=0.7606) (Figure S4a). Differences between reference-free survival curves regarding uTFD were not statistically significant (log-rank test, p=0.8563) (Figure S4b).

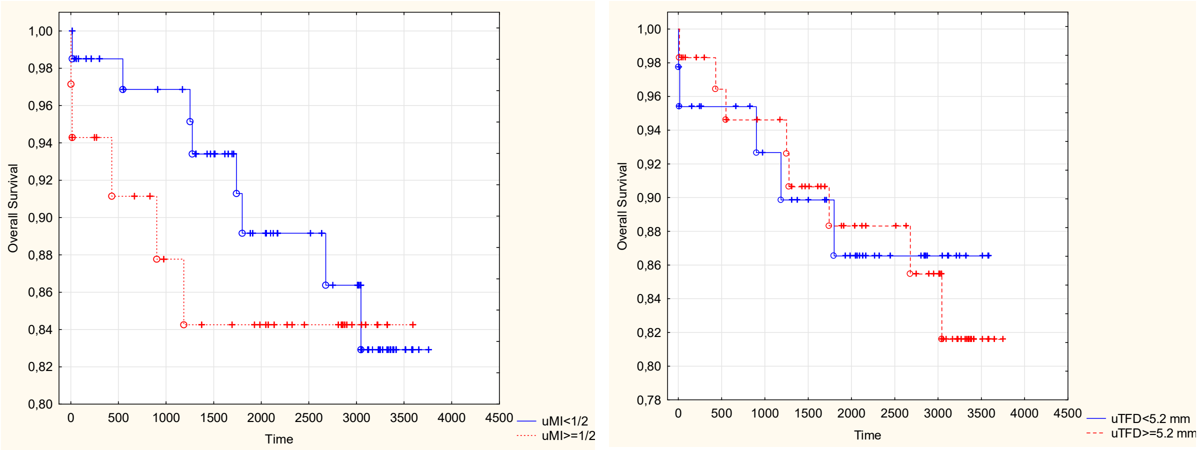

(**a**) (**b**)

Figure S3. Overall survival of patients according to uMI (**a**) and uTFD (**b**).

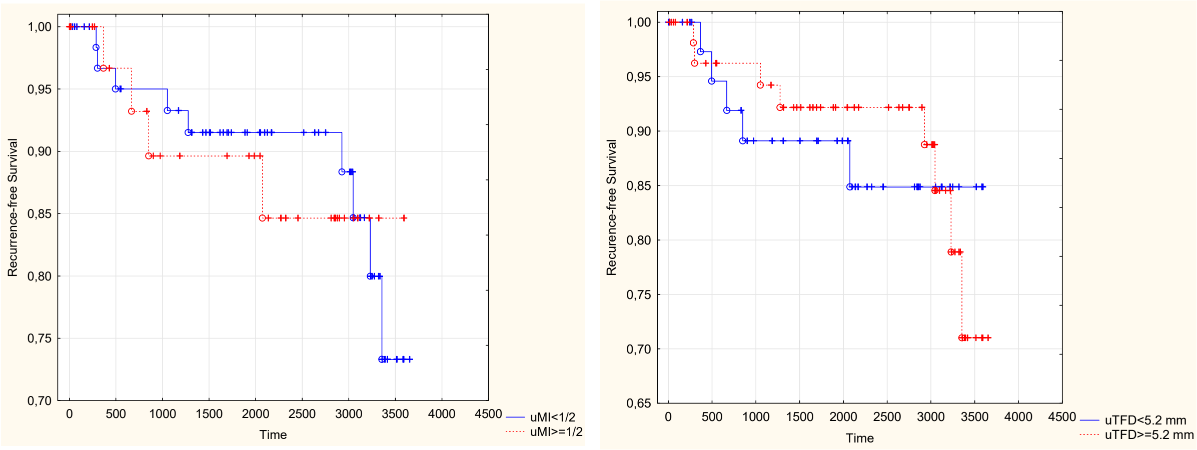

(**a**) (**b**)

Figure S4. Recurrence-free survival of patients according to uMI (**a**) and uTFD (**b**).
