# Supplementary material for: Ultrasound Measurement of Tumor-Free Distance from the Serosal Surface as the Alternative to Measuring the Depth of Myometrial Invasion in Predicting Lymph Node Metastases in Endometrial Cancer
Source: Diagnostics (Basel). 2021 Aug 14;11(8):1472. doi: 10.3390/diagnostics11081472 (PMC8392068; doi:10.3390/diagnostics11081472)
Supplement: Supplementary file 1 [file diagnostics-11-01472-s001.zip › Diagnostics_Table S1.pdf]

Table S1. Multivariate analysis of the value of predictive factors for lymph node metastasis only for patients with ESGO&ESTRO&ESP Guidelines 2021>Low (n=64 patients)*

Variable		*OR (95%CI)*	*p value*	*Significance (α=0.05)*	*ACC*	*Specificity*	*Sensitivity*	*NPV*	*PPV*
Ultrasound parameter	(u)MI (≥ 50%)	0.534 (0.148 – 1.924)	0.337	No	64.06%	65.91%	60%	78.38%	44.44%
	(u)TFD	0.943 (0.809 – 1.100)	0.456	No					

*There was no patients having metastases to the lymph nodes in the low-risk group.
