# Supplementary material for: Ultrasound Measurement of Tumor-Free Distance from the Serosal Surface as the Alternative to Measuring the Depth of Myometrial Invasion in Predicting Lymph Node Metastases in Endometrial Cancer
Source: Diagnostics (Basel). 2021 Aug 14;11(8):1472. doi: 10.3390/diagnostics11081472 (PMC8392068; doi:10.3390/diagnostics11081472)
Supplement: Supplementary file 1 [file diagnostics-11-01472-s001.zip › Diagnostics_Table S2.pdf]

Table S2. Relationship between ultrasound and histopathological measurement of the degree of endometrial cancer (MI) infiltration

	pMI		
uMI	pMI < 50%	pMI ≥ 50%	Total
uMI < 50%	60	16	76
uMI ≥ 50%	11	29	40
Total	71	45	116

Legend: uMI - ultrasound measured the depth of invasion of the uterine muscle; pMI - pathomorphologically measured the depth of invasion of the uterine muscle (Chi-square test, $p < 0.00001$).
