# Supplementary material for: Ultrasound Measurement of Tumor-Free Distance from the Serosal Surface as the Alternative to Measuring the Depth of Myometrial Invasion in Predicting Lymph Node Metastases in Endometrial Cancer
Source: Diagnostics (Basel). 2021 Aug 14;11(8):1472. doi: 10.3390/diagnostics11081472 (PMC8392068; doi:10.3390/diagnostics11081472)
Supplement: Supplementary file 1 [file diagnostics-11-01472-s001.zip › Diagnostics_Table S3.pdf]

Table S3. Kruskal-Wallis test for uTFD grouped by risk level (ESGO 2012)

	Low<br>*R: 70.953*	*Intermediate*<br>*R: 48.328*	*High*<br>*R: 45.433*
Low		0.007310	0.002424
Intermediate	0.007310		1.000000
High	0.002424	1.000000	

Legend: R – Average ranks for groups
