# Supplementary figures and images for: Ultrasound Measurement of Tumor-Free Distance from the Serosal Surface as the Alternative to Measuring the Depth of Myometrial Invasion in Predicting Lymph Node Metastases in Endometrial Cancer
Source: Diagnostics (Basel). 2021 Aug 14;11(8):1472. doi: 10.3390/diagnostics11081472 (PMC8392068; doi:10.3390/diagnostics11081472)

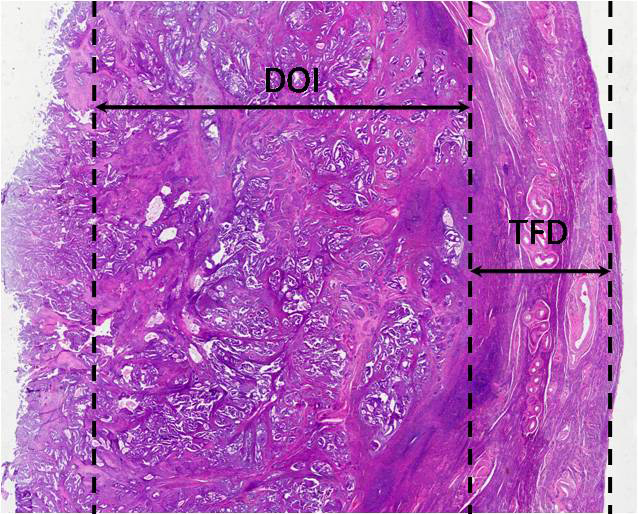

Supplement: Supplementary file 1 [file diagnostics-11-01472-s001.zip › Diagnostics_Figure S1.jpg]

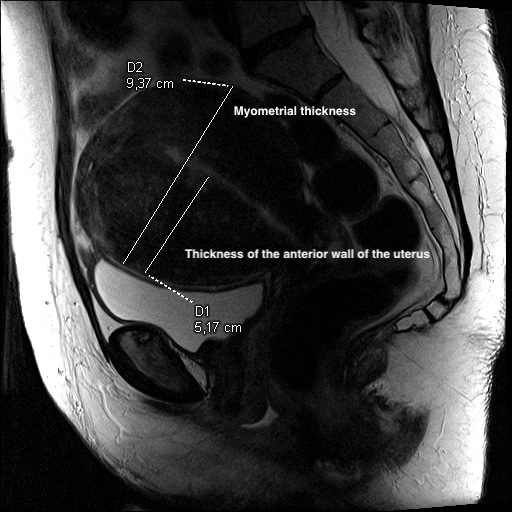

Supplement: Supplementary file 1 [file diagnostics-11-01472-s001.zip › Diagnostics_Figure S6.jpg]

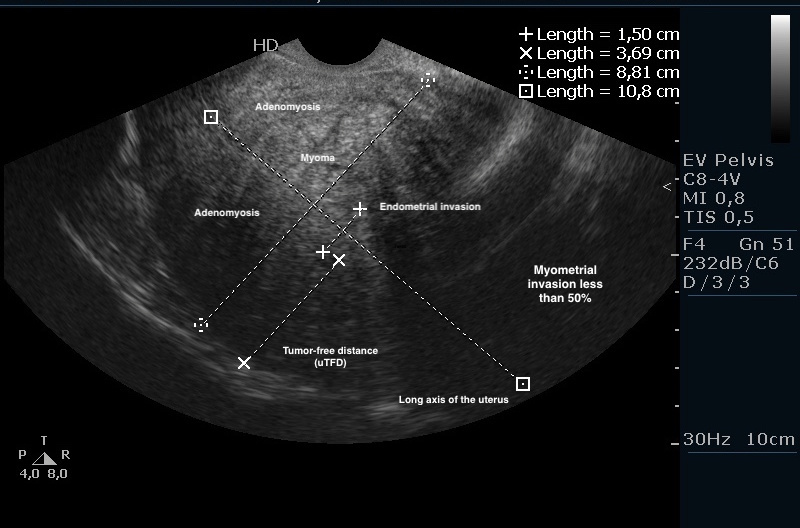

Supplement: Supplementary file 1 [file diagnostics-11-01472-s001.zip › Diagnostics_Figure S7.jpg]
